# Supplementary material for: A Critical View of the Application of the APEX Software (Aqueous Photochemistry of Environmentally-Occurring Xenobiotics) to Predict Photoreaction Kinetics in Surface Freshwaters
Source: Molecules. 2019 Dec 18;25(1):9. doi: 10.3390/molecules25010009 (PMC7017383; doi:10.3390/molecules25010009)
Supplement: Supplementary file 1 [file molecules-25-00009-s001.zip › 3.2.4_gcc-4.4.0.zip!/3.2.4_gcc-4.4.0/3.2.4_gcc-4.4.0/doc/octave/html/interpreter/Comma-Separated-Lists.html]

Comma Separated Lists - Untitled


Previous: Cell Arrays,
Up: Data Containers


---

### 6.3 Comma Separated Lists

Comma separated lists ^1^ are the basic argument type
to all Octave functions - both for input and return arguments. In the
example

```
     max (a, b)
```

a, b is a comma separated list. Comma separated lists
can appear on both the right and left hand side of an assignment. For
example

```
     x = [1 0 1 0 0 1 1; 0 0 0 0 0 0 7];
     [i, j] = find (x, 2, "last");
```

Here, x, 2, "last" is a comma separated list constituting
the input arguments of `find`. `find` returns a comma
separated list of output arguments which is assigned element by
element to the comma separated list i, j.

Another example of where comma separated lists are used is in the
creation of a new array with `[]` (see Matrices) or the
creation of a cell array with `{}` (see Basic Usage of Cell Arrays). In the expressions

```
     a = [1, 2, 3, 4];
     c = {4, 5, 6, 7};
```

both 1, 2, 3, 4 and 4, 5, 6, 7 are comma separated lists.

Comma separated lists cannot be directly manipulated by the
user. However, both structure arrays and cell arrays can be converted
into comma separated lists, and thus used in place of explicitly
written comma separated lists. This feature is useful in many ways,
as will be shown in the following subsections.

- Comma Separated Lists Generated from Cell Arrays- Comma Separated Lists Generated from Structure Arrays

---

#### Footnotes

[1] Comma-separated lists are also sometimes
informally referred to as cs-lists.

---
